# Evaluation of nosocomial infections after congenital heart surgery in children: a comprehensive analysis including the systemic immune-inflammation index

**DOI:** 10.3389/fped.2026.1735292

**Published:** 2026-05-26

**Authors:** Sercan Tak, Tuğba Bedir Demirdağ, Erkan İriz, Eda Nur Yiğiter, Issa Aden Ahmed Shide, Subhan Mammadov, Anıl Tapısız, Fatma Hayvacı Canbeyli, Mutlu Uysal Yazıcı, Ebru Azapağası

**Affiliations:** 1 Department of Cardiovascular Surgery, Gazi University Faculty of Medicine, Ankara, Türkiye; 2 Division of Pediatric Infectious Diseases, Department of Pediatrics, Gazi University Faculty of Medicine, Ankara, Türkiye; 3 Division of Pediatric Cardiology, Department of Pediatrics, Gazi University Faculty of Medicine, Ankara, Türkiye; 4 Division of Pediatric Critical Care Medicine, Department of Pediatrics, Gazi University Faculty of Medicine, Ankara, Türkiye

**Keywords:** bloodstream infection, cardiopulmonary bypass, congenital heart disease, nosocomial infections, pneumonia, systemic immune-inflammation index

## Abstract

**Introduction:**

Nosocomial infections (NIs) following cardiac surgery in children with congenital heart disease (CHD) remain a significant contributor to morbidity, mortality, and prolonged hospital stays. This study aimed to identify independent risk factors for NI and to evaluate the predictive utility of the Systemic Immune-Inflammation Index (SII) in this high-risk population.

**Material and methods:**

This was a single-center, retrospective observational study involving patients who underwent cardiovascular surgery between January 2015 and March 2025 and remained hospitalized for > 48 h. NI was defined based on the Center for Disease Control's National Healthcare Safety Network (CDC/NHSN) criteria. Multivariate logistic regression analysis using the ‘backward conditional’ method was performed to determine independent predictors of NI. Baseline and postoperative SII were calculated.

**Results:**

A total of 209 children were included in the study. NIs were observed in 46 patients (22.0%), with sepsis (69.6%) and pneumonia (30.4%) being the most frequent types. Gram-negative bacteria (58.4%) predominated. Patients with NI were significantly younger, more frequently had cyanotic CHD, comorbidities, and higher Aristotle scores. Univariate analysis showed that postoperative SII was significantly lower in the infection group (562.73 vs. 916.28; *p* < 0.001). However, multivariate analysis identified four independent predictors of increased NI risk; younger age (risk decreased by 2.2% per month: OR = 0.978, 95% CI = 0.957–0.999, *p* = 0.041), mechanical ventilation duration (risk increased by 1.2% per hour: OR = 1.012, 95% CI = 1.00–1.024, *p* = 0.047), postoperative length of stay (LOS) (risk increased by 16.2% per day: OR = 1.162, 95% CI = 1.031–1.309, *p* = 0.014), and presence of postoperative complications (risk increased∼110-fold: OR = 110.365, 95% CI = 8.042–1514.674, *p* < 0.001). Postoperative SII did not retain a strong independent predictive value in the multivariate model.

**Discussion:**

Postoperative complications are the strongest independent predictor for NIs following pediatric cardiac surgery. Prolonged mechanical ventilation and postoperative LOS significantly increase this risk. While SII correlates with infection status negatively, it does not function as a robust independent predictor in this patient population, possibly due to the overwhelming inflammatory response induced by cardiopulmonary bypass. These findings underscore the need for stringent infection control and aggressive management of postoperative morbidity.

## 1 Introduction

Children undergoing cardiac surgery may be at risk of developing nosocomial infections (1). The range varies between 2% and 48% in different studies (2, 3). Such infections contribute substantially to morbidity and mortality, necessitate increased antibiotic utilization, and result in prolonged lengths of hospital stay (2). Cardiac surgery is frequently complicated by surgical site infections (SSI), bloodstream infections (BSI), and ventilator-associated pneumonia (VAP) (4). While SSI accounts for a notable proportion of cases (2.3%–8%), more serious infections, including septicemia (6.3%–15%), mediastinitis (0.2%–3.3%), and endocarditis (0.2%), also represent significant postoperative complications (5). Well-known major risk factors for postoperative infections following cardiac surgery include younger age at surgery and complexity of the procedure (6). Other risk factors include prolonged preoperative hospitalization, elevated American Society of Anesthesiologists (ASA) classification, preoperative mechanical ventilation, extended operative time, perioperative blood transfusion, continuation of antimicrobial prophylaxis for more than 48 h, longer intensive care unit (ICU) and hospital stays, open chest management, higher surgical risk grades, and prolonged central line duration (7).

The spectrum of infections, risk factors, and etiological agents after cardiac surgery may vary depending on the center. Surgery-related infections are most frequently caused by *Staphylococcus aureus*, Coagulase-negative staphylococci (CoNS), *Escherichia coli, Enterococcus faecalis*, and *Pseudomonas* spp. Epidemiological trends over the past decade indicate a decline in Gram-negative bacilli, accompanied by a rising proportion of *S. aureus* associated infections (8).

The systemic immune-inflammation index (SII) is a novel inflammatory biomarker calculated based on the circulating counts of neutrophils, lymphocytes, and platelets. It was initially defined to determine the treatment process and predict poor outcomes in patients with hepatocellular carcinoma, and it is currently proposed that it can be used as a strong predictive tool for poor outcomes in both malignancy and inflammatory conditions (9). Some studies conducted in the adult population have demonstrated that it is predictive of poor outcomes in conditions such as heart failure, coronary artery disease, and atrial fibrillation, and that it exhibits a linear correlation with other cardiac markers (10, 11). However, studies concerning children, particularly those who have undergone congenital heart surgery, are highly limited.

The present study was conducted with the primary purpose of identifying risk factors and evaluating the predictive value of SII for hospital-acquired infections in patients undergoing congenital cardiac surgery, while secondary objectives included quantifying their prevalence and elucidating the resultant clinical outcomes.

## 2 Materials and methods

### 2.1 Study design and patient population

This retrospective observational study was conducted in Gazi University Hospital/Pediatric Cardiac Surgery Department. The study included consecutive patients (0–18 years) who underwent cardiovascular surgery between January 2015 and March 2025, provided they remained hospitalized for at least 48 h postoperatively. Patients with incomplete preoperative data, those who underwent percutaneous interventions without surgery, patients who underwent emergency surgery, and patients discharged within 48 h after surgery were excluded.

The study protocol was approved by the institutional ethics committee (Approval No: E.1265727). Given the retrospective nature of the study, the requirement for written informed consent was waived.

### 2.2 Data collection and definitions

Patient charts and records were collected using a standardized data collection form. The following variables were recorded: demographic characteristics (age, sex, body weight, percentile status), type of congenital heart disease and oxygenation status, comorbidities (respiratory, neurologic, renal, or other chronic conditions), preoperative length of hospital stay (days), preoperative laboratory findings (C-reactive protein (CRP), aspartate transaminase (AST), alanine transaminase (ALT), white blood cells (WBC), neutrophils, lymphocytes, and platelets), perioperative data [cardiopulmonary bypass (CPB) duration, cross-clamp (X-clamp) time], transfusion status and volume (units), duration of mechanical ventilation (hours), length of intensive care unit (ICU) and hospital stay (days), and postoperative complications [low cardiac output syndrome (LCOS), extubation failure, extracorporeal membrane oxygenation (ECMO) requirement, etc].

Cyanotic congenital heart disease constitutes a heterogeneous spectrum of structural abnormalities affecting the development of the heart and great intrathoracic vessels. The resulting cyanosis typically arises from mechanisms such as obstruction to pulmonary blood flow, unfavorable intracardiac streaming, or the complete mixing of systemic and pulmonary venous returns alongside decreased pulmonary blood flow (12). The Aristotle Scoring System is a risk stratification tool that evaluates congenital cardiac surgical procedures based on mortality, morbidity, and surgical difficulty levels, classifying these procedures into 4 levels according to their complexity (13).

### 2.3 Definition of nosocomial infections and microbiology

Nosocomial infection (NI) was defined as an infection occurring > 48 h after hospitalization and confirmed by clinical findings together with laboratory, microbiological, or radiological evidence. Definitions were based on the Center for Disease Control's National Healthcare Safety Network (CDC/NHSN) criteria (e.g., SSI, BSI, VAP) (14). Positive cultures were considered as true pathogens if consistent with clinical findings; single, potentially contaminant isolates were excluded. Identified pathogens were categorized as Gram-positive or Gram-negative.

Blood, urine, sputum/endotracheal aspirate, and wound samples were processed on standard media, and organisms were identified with automated systems (e.g., VITEK/BD Phoenix or equivalent). Antibiotic susceptibility testing was performed according to European Committee on Antimicrobial Susceptibility Testing (EUCAST) guidelines. Laboratory parameters (CRP, renal and liver function tests, leukocyte count, neutrophil count, lymphocyte count, platelet count) were recorded from samples obtained preoperatively or within 24–48 h postoperatively.

### 2.4 Perioperative variables and definitions

CPB and cross-clamp durations were recorded in minutes from operative records. Mechanical ventilation time was calculated as total hours from intubation to extubation. Transfusion was documented as presence/ absence and total number of units. Postoperative complications were defined as clinically significant adverse events requiring medical or surgical intervention (e.g., LCOS, ECMO support, extubation failure, acute renal failure). The SII was calculated for each patient using parameters measured both at the day of hospital admission and on the first postoperative day. It was first defined by Hu et al. (9) in 2014 as an easily accessible and highly prognostic biomarker calculated using the formula *SII = platelet count* *×* *neutrophil count/lymphocyte count*. This index highlights the critical interplay among neutrophils, lymphocytes, and platelets in inflammatory responses.

### 2.5 Statistical analysis

Data were analyzed using Statistical Package for Social Sciences (SPSS, version 27, IBM, USA). Descriptive statistics included number, percentage, mean ± standard deviation (SD), minimum, and maximum. Normality of continuous variables was assessed using both visual (histograms, probability plots) and analytical methods (Kolmogorov–Smirnov test). Non-normally distributed continuous variables were compared using the Mann–Whitney U test, while categorical variables were compared using the chi-square test or Fisher's exact test. Continuous data were primarily presented as median (IQR), and categorical data as percentages. Multivariate logistic regression analysis was performed to determine independent predictors of NIs. Potential significant variables were evaluated step-by-step using the ‘backward conditional’ method. The following were used as covariates: age, Aristotle score, CRP, WBC, preoperative length of stay (LOS), CPB time, X-clamp time, ICU time, postoperative LOS, SII-postoperative, mechanical ventilation time, postoperative complication, oxygenation, and comorbidity. Of these, postoperative complications, oxygenation, and comorbidity were treated as categorical covariates. The results of the logistic regression models were presented as odds ratio (OR) with their corresponding 95% confidence interval (CI). The model fit was assessed by Hosmer-Lemeshow goodness-of-fit statistics. A *p*-value ≤0.05 was considered statistically significant.

## 3 Results

### 3.1 Patient characteristics and perioperative findings

**Figure 1:**
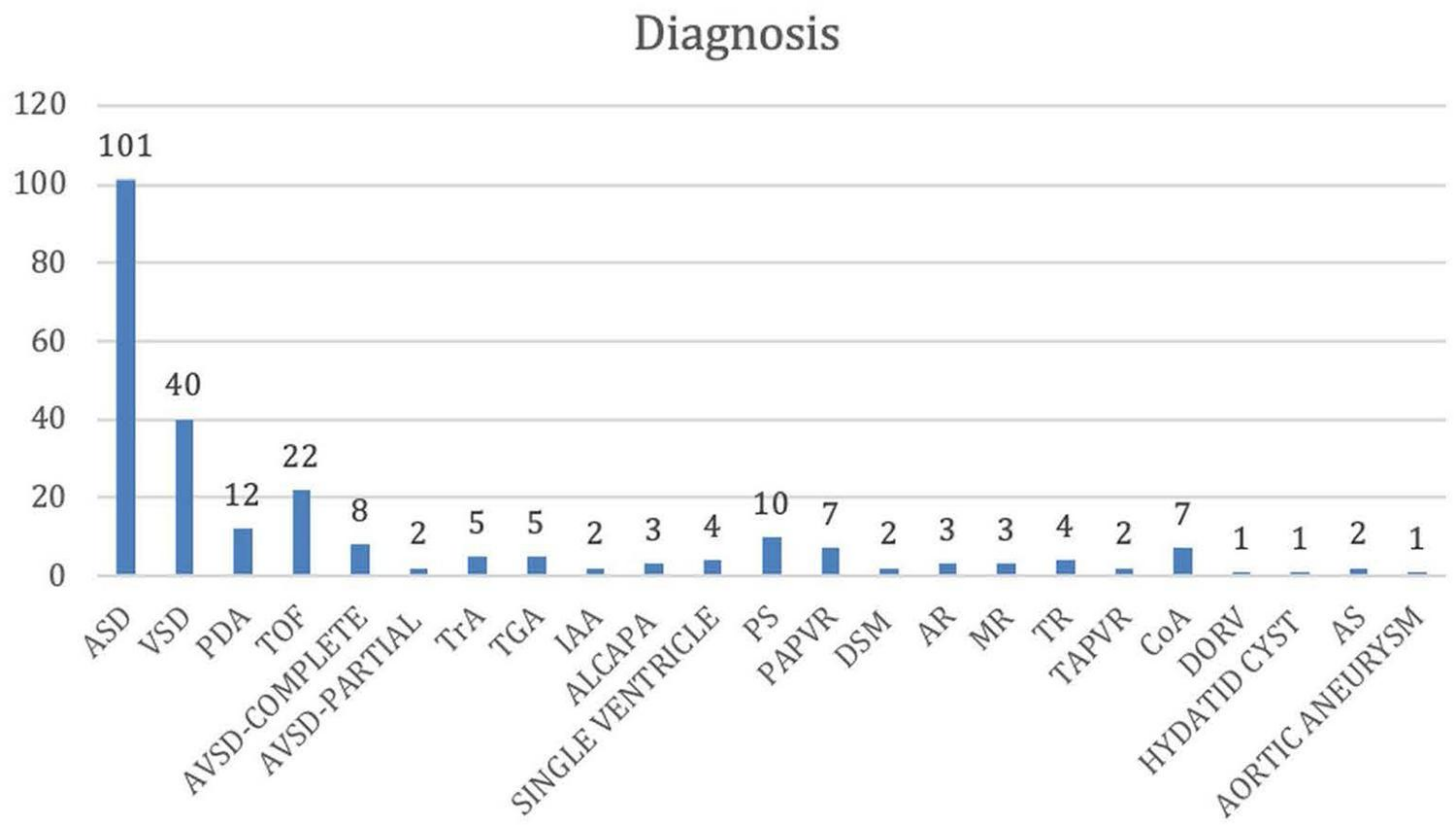
Detailed diagnostic distribution of the study cohort comprising 209 children undergoing cardiovascular surgery. ASD, atrial septal defect; VSD, ventricular septal defect; PDA, patent ductus arterious; TOF, tetralogy of fallot; AVSD, atrioventricular septal defect; TrA, tricuspid atresia; TGA, transposition of great arteries; IAA, interrupted aortic arch; PS, pulmonary stenosis; PAPVR; partial anomalous pulmonary venous return; DSM, discrete subaortic membrane; AR, aortic regurgitation; MR, mitral regurgitation; TR, tricuspid regurgitation; TAPVR, total anomalous pulmonary venous return; CoA, coarctation of aorta; DORV, double outlet right ventricle; AS, aortis stenosis.

A total of 209 patients who underwent cardiovascular surgery were included in the study (Table 1). Of these, 51.2% were male and 48.8% were female. The mean (± SD) age was 57.40 ± 49.04 months. The mean body weight of patients was 18.17 ± 14.24 kg, with 25.4% of patients below the 3rd percentile and 10% between 3 and 10 percentile. Cyanotic heart disease was present in 19.6% of patients, and 21.5% had comorbidities. The major underlying diseases were; Down syndrome in 35.5% (*n* = 16), other genetic disorders in 11.1% (*n* = 5), and prematurity in 15.6% (*n* = 7). 14.8% of the patients had more than one cardiac anomaly. 31.1% of patients had a history of cardiac surgery. 101(48.3%) of the patients underwent surgery for atrial septal defect (ASD), 40(19.1%) for ventricular septal defect (VSD), and 22(10.5%) for Tetralogy of Fallot (TOF). The distribution of cardiac diseases is shown in Figure 1.

**Table 1:** Baseline demographic and clinical characteristics of the study cohort comprising 209 children who underwent cardiovascular surgery.

Variables		Value
Sex (n) (%)	Male	107 (51.2)
	Female	102 (48.8)
Age (months)		57.40 ± 49.04
Body weight (kg)		18.17 ± 14.24
Percentile ranges (n) (%)	<3	53 (25.4)
	3–10	21 (10.0)
	10–25	38 (18.2)
	25–75	68 (32.5)
	>75	29 (13.9)
Oxygenation (n) (%)	Cyanotic	41 (19.6)
	Acyanotic	168 (80.4)
Presence of comorbidities (n) (%)		45 (21.5)
Comorbid factor (*n* = 45) (n) (%)	History of cardiac surgery	14 (31.1)
	Down syndrome	11 (24.4)
	Prematurity	7 (15.6)
	Genetic disorders	5 (11.1)
	Down syndrome + others	5 (11.1)
	Metabolic disorders	2 (4.4)
	Gastrointestinal anomalies	1 (2.2)
Cardiac anomaly (n) (%)	Single	178 (85.2)
	Multiple	31 (14.8)

Data are presented as mean ± sd for continuous variables, and numbers and percentages for categorical variables.

### 3.2 Nosocomial infections

**Figure 2:**
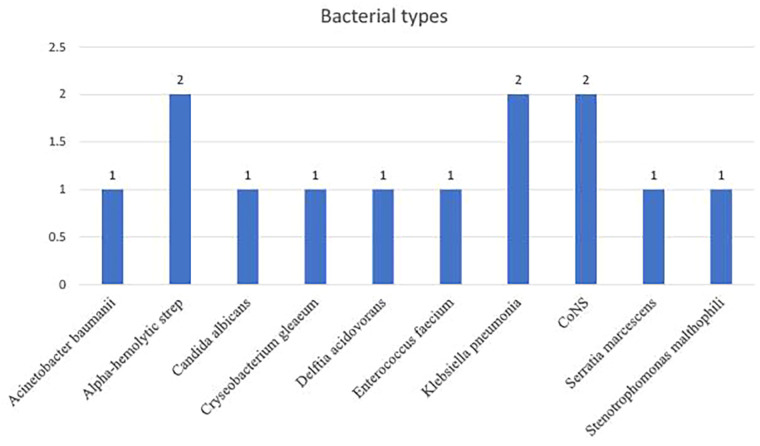
Microbiological profile and distribution of pathogens isolated from patients who developed nosocomial infections following cardiovascular surgery. CoNS, coagulase-negative staphylococci.

NIs were observed in 22% of patients. Among the 46 patients with infections, 28.3% had microbiologically confirmed infections. 69.6% had nosocomial sepsis, 30.4% had pneumonia. Of the 12 identified bacterial pathogens, 41.6% were Gram-positive and 58.4% were Gram-negative (Figure 2).

### 3.3 Comparison of patient characteristics by infection status

Patients with NIs were significantly younger than those without (*p* < 0.05). The infection rate was significantly higher in patients with cyanotic heart disease (46.3% vs. 16.1%), in patients with comorbidities (46.7% vs. 15.2%), and in patients with higher Aristotle scores (*p* < 0.05). No significant differences were observed regarding gender or number of anomalies. A body weight below the 10th percentile was significantly associated with an increased risk of NI (*p* < 0.05) (Table 2).

**Table 2:** Comparison of baseline demographic, clinical, and preoperative laboratory parameters between the patients with and without nosocomial infections.

Variables		NI Negative *n* = 163	NI Positive *n* = 46	Total *n* = 209	*P* value
Sex (n) (%)	Male	86 (80.4)	21 (19.6)	107 (51.2)	0.394
	Female	77 (75.5)	25 (24.5)	102 (48.8)	
Age (months), median (IQR)		66 (24–102)	7.75 (2.62–22)	54 (13–96)	**<0** **.** **001** ^a^
Oxygenation (n) (%)	Cyanotic	22 (53.7)	19 (46.3)	41 (19.6)	**<0** **.** **001** ^b^
	Acyanotic	141 (83.9)	27 (16.1)	168 (80.4)	
Quantity of anomaly (n) (%)	Single	137 (77.0)	41 (23.0)	178 (85.2)	0.392
	Multiple	26 (83.9)	5 (16.1)	31 (14.8)	
Presence of comorbidity (n) (%)	No	139 (84.8)	25 (15.2)	164 (78.5)	**<0** **.** **001** ^b^
	Yes	24 (53.3)	21 (46.7)	45 (21.5)	
Body weight <10p (n) (%)		46 (28.2)	28 (60.8)	74 (35.4)	**<0** **.** **001** ^b^
Aristotle score, median (IQR)		1 (1–2)	3 (2–3)	2 (1–2)	**<0** **.** **001** ^a^
SII-preoperative, median (IQR)		295.85 (185.64–475.03)	281.33 (155.92–407.70)	388.55 (54.56–1,921.28)	0.551
CRP mg/dL, median (IQR)		1.93 (1.25–2.89)	2.38 (1.48–4.80)	1.99 (1.28–3.01)	**0** **.** **025** ^a^
AST U/L, median (IQR)		34 (26–43.7)	40 (28.7–52.7)	35 (26–45)	**0** **.** **015** ^a^
ALT U/L, median (IQR)		16 (12–20)	19.5 (12.7–28)	17 (12–22)	**0** **.** **029** ^a^
Creatinine (mg/dL), median (IQR)		0.37 (0.28–0.44)	0.32 (0.24–0.47)	0.36 (0.27–0.44)	0.273
WBC x10^3^/µL, median (IQR)		8.7 (6.96–10.7)	10.53 (7.97–13.28)	9.0 (7.19–11.25)	**0** **.** **004** ^a^
Lymphocyte x10^3^/µL, median (IQR)		3.60 (2.70–5.33)	4.45 (3.19–6.89)	3.66 (2.78–5.61)	0.050
Neutrophil x10^3^/µL, median (IQR)		3.30 (2.62–5.10)	3.41 (2.57–5.52)	4.03 (2.6–5.21)	0.628
Platelet x10^3^/µL, median (IQR)		320 (269–384)	323.5 (251.7–401.0)	321 (264.5–390)	0.834

Data are presented as median (IQR) for continuous variables, and as numbers and percentages for categorical variables. SII, Systemic immune-inflammation index; NI, Nosocomial infection.

a Mann-whitney U test.

b Chi-square test (or fisher's exact test, as appopriate).

Regarding congenital heart defect types, patients without NIs had a significantly higher rate of ASD (57.7% vs. 15.2%), whereas patients with infections had higher rates of complete atrioventricular septal defect (AVSD) (10.9%) and transposition of great arteries (TGA) (10.9%) compared to those without infections (1.8% and 0%, respectively; *p* < 0.05).

### 3.4 Preoperative laboratory findings and NIs

Patients with NIs had significantly higher CRP, AST, ALT, and WBC levels compared to those without infections (*p* < 0.05). No significant differences were observed for other laboratory parameters, including median SII-preoperative (*p* > 0.05) (Table 2).

### 3.5 Perioperative factors and NIs

Preoperative hospitalization, cross-clamp time, ICU stay, CPB duration, mechanical ventilation duration, and total hospital stay were significantly longer in patients with NIs (*p* < 0.05). Postoperative complications were observed in 97.8% of patients with infections vs. 15.3% of those without, with a statistically significantly higher rate in the infection-positive group (*p* < 0.05). Conversely, there was no significant association between transfusion and NIs (Table 3). SII on the first post-operative day was 916.28 (575.38–1318) in patients who did not have NIs, whereas 562.73 (459.76–910.35) in patients who had NI (*p* < 0,001) (Table 3).

**Table 3:** Comparison of perioperative surgical data and postoperative clinical outcomes between the patients with and without nosocomial infections.

Variables		NI Negative *n* = 163	NI Positive *n* = 46	Total *n* = 209	*P* value
Preoperative LOS (day), median (IQR)		2 (1–3)	5 (2–9)	2 (2–4)	<0.001^a^
X-clamp time (minute), median (IQR)		33 (21–51)	71 (45–98)	37 (22–68)	<0.001^a^
CPB time (minute), median (IQR)		53 (39–78)	101 (67.5–136.2)	58 (40–96)	<0.001^a^
Transfusion (n) (%)	No	19 (73.1)	7 (26.9)	26 (12.4)	0.518
	Yes	144 (78.7)	39 (21.3)	183 (87.6)	
Amount of transfusion (unit), median (IQR)		2 (2–3)	2,5 (2–4)	2 (2–3)	0.051
SII-postoperative, median (IQR)		916,28 (575,38–1,318)	562, 73 (459,76–910,35)	841,04 (536,5–1,224,7)	<0.001^a^
Mechanical ventilation time (hour), median (IQR)		3 (2–17)	94 (28–216)	4 (2–28)	<0.001^a^
ICU LOS (day), median (IQR)		1.63 (0,88–2,0)	12 (5.75–24.50)	1.83 (0.92–5.5)	<0.001^a^
Postoperative complication (n) (%)		25 (15.3)	45 (97.8)	70 (33.5)	<0.001^b^
Postoperative LOS (day), median (IQR)		6 (4–7)	22.5 (15–37)	6 (5–14.5)	<0.001^a^

Data are presented as median (IQR) for continuous variables, and as numbers and percentages for categorical variables. SII, Systemic immune-inflammation index; LOS, length of stay; ICU, intensive care unit; CPB, cardiopulmonary bypass; NI, Nosocomial infection.

a Mann-whitney U test.

b Chi-square test (or fisher's exact test, as appopriate).

Postoperative complications occurred in 33.5% of patients. Among the 70 patients with complications, 40% had LCOS, 17.1% required ECMO, and acute renal failure developed in 14.3% and atrioventricular block developed in 12.9% of these patients (Table 4).

**Table 4:** Distribution and frequency of specific postoperative complications in the study cohort of 209 patients following cardiovascular surgery.

Presence of postoperative complication (n) (%)	70 (33,5)	
Complication	*n* = 70	%
Atelectasis	8	11.4
AV Block	9	12.9
Pleural effusion	6	8.6
Pneumothorax	5	7.1
ECMO	12	17.1
LCOS	28	40.0
Peritoneal dialysis	10	14.3
Re-intubation	2	2.9
Pulmonary hypertension	6	8.6
SIRS	2	2.9
CVVHD	4	5.7
Reoperation due to bleeding	2	2.9
Neurological event	6	8.6
Arrhythmia	3	4.3
Delayed sternal closure	1	1.4

Data are presented as numbers and percentages. AV, atrioventricular; ECMO, extracorporeal membrane oxygenation; LCOS, low cardiac output syndrome; SIRS, systemic inflammatory response syndrome; CVVHD, continuous venovenous hemodialysis.

### 3.6 Risk factors for NIs

The regression analysis of variables affecting the risk of NI is summarized in Table 5. Multivariate logistic regression analysis revealed that increasing age was associated with a 2.2% decrease in risk (OR = 0.978, 95% CI = 0.957–0.999, *p* = 0.041). Furthermore, each additional hour of mechanical ventilation increased the risk by 1.2% (OR = 1.012, 95% CI = 1.000–1.024, *p* = 0.047); each additional day of postoperative LOS was associated with a 16.2% increase in risk (OR = 1.162, 95% CI = 1.031–1.309, *p* = 0.014); and the presence of postoperative complications increased the infection risk 110.365-fold (OR = 110.365, 95% CI = 8.042–1514.674, *p* < 0.001).

**Table 5:** Multivariate logistic regression analysis using the backward stepwise elimination method to identify independent predictors of nosocomial infections in children following cardiovascular surgery: results presented as adjusted odds ratios (ORs) and 95% confidence intervals (CIs).

Variables	Nosocomial infection		
	Odds ratio	95% confidence interval	*P* value
Age	0.978	(0.957–0.999)	**0** **.** **041**
Mechanical ventilation time	1.012	(1.00–1.024)	**0** **.** **047**
Postoperative LOS	1.162	(1.031–1.309)	**0** **.** **014**
Postoperative complication^a^	110.365	(8.042–1514.674)	**<** **0.001**

Variables with superscript ^a^ are selected as categorical co-variate.

LOS, length of stay.

## 4 Discussion

NIs pose a significant challenge in the postoperative recovery of children with congenital heart diseases (CHD), serving as a major contributor to both morbidity and mortality following cardiac surgery (3, 15). Identifying specific risk factors is essential for recognizing high-risk patient populations and developing targeted prevention strategies.

In this study, the overall NI rate was 22.0%, while the culture-proven rate was 6.2%, with sepsis and pneumonia being the most frequent manifestations. No SSIs were observed. These findings align with the reported literature range of 13% to 30% following pediatric cardiac surgery (16). The discrepancy between clinical diagnosis and microbiological confirmation may stem from severe postoperative inflammation, which can mimic NI criteria and potentially lead to overdiagnosis. Consequently, these results emphasize the necessity for prospective validation and rigorous monitoring of patients in the postoperative period.

Our study confirms that younger age is a significant predictor of NIs, with younger children experiencing higher infection rates than older age groups. This finding is consistent with established literature identifying younger age as a key risk factor for postoperative complications (17), as well as bacterial infections following pediatric heart transplantation (18).

While obesity is a recognized risk factor for infections in adult cardiac surgery (19, 20), our findings emphasize the impact of undernutrition in the pediatric population. Specifically, we observed that NIs occurred more frequently in children with a body weight below the 10th percentile. This suggests that a weight below this specific threshold, rather than general undernourishment (15), may be a critical determinant of infection risk. These results underscore the clinical necessity of targeted nutritional support to maintain body weight above the 10th percentile. We believe this observation provides a specific benchmark for the literature and warrants further validation in prospective studies.

Comorbidities significantly increased the frequency of NIs in our cohort. However, contrary to the findings of Sen et al. (15), an increase in the number of cardiac anomalies did not alter the risk of NIs in this study. This suggests that the presence of non-cardiac comorbidities may be a more potent predictor of infection than the anatomical complexity of the heart disease itself.

NIs were more prevalent in children with cyanotic heart disease than in those with acyanotic conditions. This aligns with previous reports indicating that hypoxia (oxygen saturation < 85%) and cyanosis are associated with increased infection rates (1, 15). This vulnerability likely stems from impaired tissue oxygenation, which compromises host defense mechanisms. Additionally, cyanosis is often associated with higher cardiac complexity scores, reflecting a more significant surgical and physiological burden.

Although perioperative blood product transfusion has been linked to increased NI incidence (21), our study found no significant association between transfusion volume or duration and infection rates. This is consistent with other pediatric cohorts where transfusion was not an independent risk factor after adjusting for clinical variables (16). Discrepancies between studies likely reflect variations in patient populations, surgical complexity, and institutional transfusion thresholds. These results suggest that the impact of transfusion on infection risk is multifactorial and requires further large-scale prospective investigation (21, 22).

In our series, nearly all patients diagnosed with a NI had also experienced at least one postoperative complication (*n* = 45/46, 97.8%). This clinical overlap explains the high odds ratio observed in our model, as the presence of complications necessitates prolonged use of invasive instruments, such as central venous catheters, endotracheal tubes, and urinary catheters, which are well-established risk factors for infection. Furthermore, major invasive interventions required to manage these complications, including ECMO, peritoneal dialysis, and reoperation for bleeding, significantly increase the risk of infectious exposure. Given that these complications typically manifested in the very early postoperative period, followed by NI diagnosis several days later, it is reasonable to suggest that the observed NI rates were a consequence of postoperative morbidity rather than the surgical procedure itself. This highlights the critical importance of both preventing complications through meticulous intensive care management and adhering to stringent infection control protocols in the postoperative period.

In our multivariate logistic regression analysis, postoperative complications emerged as a highly significant predictor of nosocomial infection (OR: 110.365; 95% CI: 8.042–1514.674; *p* < 0.001). The exceptionally high OR and the wide CI associated with this variable likely reflect a strong clinical association and temporal overlap between the development of complications and the onset of infection. Mathematically, this extreme OR value may stem from a near-perfect distribution within the cohort; specifically, if the incidence of complications is near zero in the non-infection group or present in almost all patients with infection, the logistic regression algorithm identifies the variable as a near-certain predictor, causing the OR to trend toward infinity. Furthermore, the presence of preoperative risk factors, such as advanced New York Heart Association (NYHA) class and systemic venous congestion in our high-risk cohort, may have further predisposed these patients to both complications and subsequent infections. Therefore, this finding should be interpreted as a powerful clinical association rather than a strictly unidirectional causal relationship.

In our multivariate regression analysis, prolonged mechanical ventilation was identified as a significant risk factor for NI, a finding that is well-supported by numerous studies and remains a predictable clinical outcome. Although prolonged LOS also appeared as a statistically independent risk factor, it would be inappropriate to categorize this as a primary driver of NI. In such a fragile patient population undergoing major cardiac surgery, managing these infections is an exceptionally challenging and protracted process. Therefore, the extended LOS observed in patients with NI should be interpreted as a consequence of the infection and the complex recovery process rather than a pre-existing risk factor. Our results underscore that while certain perioperative variables predispose patients to NI, the resulting infections further complicate the clinical course, leading to the inevitably prolonged hospitalization observed in our cohort.

While the SII is an established prognostic biomarker in oncology and cardiovascular diseases, its role in predicting infectious outcomes remains less explored. High SII levels have been linked to increased mortality in patients with sepsis (23), though other data suggest a more complex, inverted J-shaped association with 28-day mortality (24). These conflicting or limited findings in infectious cohorts highlight the need to evaluate SII specifically within the context of postoperative recovery in pediatric cardiac surgery.

Research on the SII in pediatric infections remains scarce. While Walian et al. (25) identified SII as a strong predictor of poor outcomes in children undergoing CPB for acyanotic CHD, they did not specifically investigate its relationship with infection. Interestingly, while most literature associates elevated SII with adverse clinical outcomes, our study demonstrated a negative correlation between SII and the risk of early postoperative infections in children following CHD surgery.

The only other large-scale study examining the relationship between SII and early NIs following congenital heart surgery is by Li et al. (26). Consistent with our results, they reported a negative correlation, attributing this to the use of postoperative day 1 SII values; in contrast, studies reporting a positive correlation typically utilized preoperative values. While we also evaluated SII at hospital admission, we found no significant association between preoperative levels and postoperative NIs.

CPB triggers a systemic inflammatory response characterized by early and late phases (27). The interaction of blood with the CPB circuit activates the complement system and cellular components, particularly neutrophils, which play a central role in subsequent tissue damage (28, 29). Conversely, lymphocyte counts typically decrease and remain low through the first postoperative week (27). CPB also significantly affects platelets, key players in both coagulation and immuno-inflammation, reducing their count by up to 50% through mechanisms such as hemodilution, mechanical destruction, and surface adhesion (27). These CPB-induced alterations in neutrophil, lymphocyte, and platelet dynamics directly influence the SII, providing a physiological basis for its relevance in the early postoperative period.

The complex immune response triggered by CPB may result in atypical inflammatory profiles when an infection is superimposed. Specifically, the recruitment of neutrophils to infection sites, mediated by interactions between activated platelets and neutrophils, can lead to a reduction in their circulating counts (26). As suggested by Li et al., this sequestration mechanism likely explains the lower SII values observed in our cohort among patients who developed postoperative infections.

Although the postoperative SII was found to be significantly lower in patients who developed infection in our study, the multivariate analysis indicated that it lacked a strong predictive value. This discrepancy may be attributed to several different reasons. First, the relatively small sample size and inherent heterogeneity between groups may have limited the statistical power to detect SII as an independent predictor. Second, when evaluated alongside more dominant risk factors such as ‘postoperative complications’ and ‘prolonged mechanical ventilation,’ the marginal predictive contribution of SII may have been attenuated by the overwhelming impact of these clinical variables. Most importantly, CPB is associated with a complex and often deleterious systemic inflammatory response. While inflammation represents a protective effort against foreign insults, an exaggerated response during major surgery and prolonged anesthesia can lead to significant tissue damage. CPB, by its non-physiological nature, specifically the exposure of blood to non-endothelial pump surfaces and oxygenators, exacerbates this reaction. Significant hemodilution during CPB leads to fluid shifts between compartments and the dilution or denaturation of essential plasma proteins. Furthermore, exposure to artificial surfaces and abnormal shear stress triggers the activation of blood elements, the overproduction of vasoactive mediators, and the disruption of the coagulation cascade. In summary, these factors overwhelm homeostatic mechanisms, resulting in a complex and unpredictable systemic inflammatory response. Since CPB-induced general inflammation occurred in all patients, including those who did not develop a NI, this background inflammatory noise likely affected SII levels across the entire cohort. Consequently, the ability of this biomarker to independently discriminate infection-specific inflammation from CPB-induced systemic inflammation was weakened at the multivariate level.

Pediatric cardiac surgery reports on NI etiology are limited, but our findings confirm a predominance of Gram-negative bacteria. While geographical trends often show Gram-negative organisms are more common in developing countries and Gram-positive bacteria in developed nations (8), a key highlight of our study is the emergence of opportunistic pathogens. In addition to *Pseudomonas aeruginosa* and *Klebsiella* spp., we identified agents such as *Acinetobacter* spp., *Stenotrophomonas maltophilia*, *Delftia corrodens*, and *Chryseobacterium* spp. as causative factors. These pathogens, often difficult to culture and characterized by distinct antimicrobial susceptibility patterns, necessitate heightened clinical awareness and targeted surveillance in the postoperative period.

This study has several limitations that should be acknowledged. First, its retrospective design may have introduced selection and information bias. Second, the study was conducted at a single center, which may limit the generalizability of the findings to other institutions with different patient populations, surgical protocols, or infection control practices. Third, the relatively small sample size may have limited the statistical power to detect associations between certain risk factors and NIs. Fourth, although possible NIs were defined according to CDC/NHSN criteria, the potential for overdiagnosis cannot be excluded due to the presence of severe postoperative inflammatory responses. Finally, microbiological confirmation was available in only a subset of cases, which may underestimate the true prevalence and diversity of causative pathogens. Future prospective, multicenter studies are warranted to address these limitations and validate the findings.

## 5 Conclusion

Postoperative NIs remain a significant complication in pediatric cardiac surgery, with younger age, cyanotic heart disease, and underlying comorbidities identified as notable risk factors. Optimal nutritional status and careful critical care management implemented to avoid postoperative complications are also protective against infection. Although it was not a strong predictor according to multivariate analysis, a significant negative correlation exists between postoperative SII and NI. Our study also highlights the emergence of opportunistic pathogens, alongside frequently encountered Gram-negative bacteria, emphasizing the need for clinician vigilance due to variable antimicrobial susceptibility. These findings contribute to the existing literature and underscore the necessity for further prospective, multicenter studies to clarify risk factors and inform effective preventive strategies.

## Data Availability

The raw data supporting the conclusions of this article will be made available by the authors, without undue reservation.
